# Characterization of the *Trichomonas vaginalis *surface-associated AP65 and binding domain interacting with trichomonads and host cells

**DOI:** 10.1186/1471-2180-7-116

**Published:** 2007-12-25

**Authors:** Ana F Garcia, JF Alderete

**Affiliations:** 1 Department of Microbiology, University of Texas Health Science Center, 7703 Floyd Curl Drive, San Antonio, TX 78229-3900, USA; 2 School of Molecular Biosciences, Fulmer Hall, Washington State University, Pullman, WA 99164-4660, USA

## Abstract

**Background:**

AP65 is a prominent adhesin of *Trichomonas vaginalis *that mediates binding of parasites to host vaginal epithelial cells (VECs). AP65 with no secretion signal sequence, membrane targeting peptide, and anchoring motif was recently found to be secreted.

**Results:**

We first wanted to demonstrate surface association of AP65 to the parasite followed by the identification of the binding epitope interacting with both organisms and VECs. AP65 was found to bind to trichomonads, but not to trypsin-treated parasites, in an auto-ligand assay, suggesting the existence of a surface protein associating with AP65. Since rabbit antiserum IgG antibodies reactive with epitopes localized to the N-terminal region of AP65 inhibit the attachment of live parasites to VECs, we hypothesized that the binding domain was localized to this region. We subcloned five overlapping fragments of AP65 called c1 through c5, and expression of recombinant clones was confirmed with antibodies to AP65. Each purified recombinant protein was then tested for binding activity using an established ligand assay, and fragment c1 with the first twenty-five amino acids in the N-terminal domain was required for binding to VECs and, surprisingly, also to parasites. Importantly, c1 competed with the binding of AP65 to both cells types.

**Conclusion:**

*T. vaginalis *AP65 is a secreted, surface-associated protein and a model is proposed to explain how this secreted protein functions as an adhesin.

## Background

*Trichomonas vaginalis *causes trichomonosis, the most common, non-viral sexually transmitted infection (STI) in humans [1]. This STI poses a risk for adverse health consequences in both women and men. Adverse pregnancy outcomes, cervical neoplasia, atypical pelvic inflammatory disease are serious adverse outcomes for women [2-4]. Complications related to trichomonal infection in men are non-gonoccocal urethritis, prostatitis, epydidymitis, urethral disease, and infertility [4-8], and recently a relationship between trichomonosis and prostate cancer has been shown [9]. Furthermore, increased risk for HIV acquisition and seroconversion has been well documented in both women and men [10-13].

*T. vaginalis *has increased ability to cytoadhere to epithelial versus fibroblast cells [14], and it is now accepted that preparatory to successful host infection and pathogenesis is adhesion of *T. vaginalis *to vaginal epithelial cells (VECs) [15-21]. Five different adhesin proteins (AP120, AP65, AP33, AP51, and AP23) mediate adherence to VECs, are members of multigene families, and except for one AP51 gene, are coordinately up-regulated by iron in medium, lactoferrin-iron, and heme-iron [15,18,19,22-31]. Interestingly, laboratory-adapted *T. vaginalis *isolates synthesize lower amounts of adhesins and have lost the ability to up-regulate synthesis by iron [29]. Fresh clinical isolate trichomonads immediately upon adherence to VECs but not HeLa cells display a dramatic change in morphology concomitant with synthesis and surface placement of adhesins [17], and this was more recently confirmed by analysis of the numerous trichomonad genes up-regulated upon contact with VECs [20]. The adhesins AP120, AP65, AP33, and AP51 were found to have sequence identity to metabolic enzymes [19,24-28,31], which reside within the hydrogenosome [32].

AP65 is the hydrogenosomal NAD-dependent decarboxylating malic enzyme and is a prominent trichomonad adhesin [18]. This conclusion is based on several lines of evidence. First, there is a direct relationship between the amount of AP65 bound to VECs and levels of adherence compared to other adhesins [18]. Second, polyamine depletion increased levels of adherence up to 20-fold, and most of this increased adherence was abrogated by anti-AP65 antibody [21]. Third, the genetic approaches involving antisense decreased expression of *ap65 *[16] and heterologous expression of *T. vaginalis *AP65 in *T. foetus *[23] reaffirmed the role of AP65 in adherence. More recently analysis of the proteins secreted during growth of *T. vaginalis *parasites revealed numerous metabolic enzymes, among which included AP65. It is noteworthy that AP65 of the secreted protein preparation was found capable of being internalized by VECs, and this resulted in signaling of VECs for expression of genes, which included IL-8 and COX-2 [33]. Episomal expression of AP65 within epithelial cells confirmed a role for AP65 within cells in up-regulating expression of genes [33]. Therefore, as AP65 appears to play a role in establishment of infection and host response and given the significance of trichomonosis as a major STI, it is necessary to continue to characterize this adhesin and identify receptor-binding epitopes for possible future interference strategies.

In previous work, we have demonstrated that anti-AP65 serum IgG antibodies inhibit adherence of live parasites. Further, the mapping of the antibody-binding epitopes of AP65 revealed a cluster of epitopes at the amino terminus of AP65 [18]. Therefore, we hypothesized that the receptor-binding epitope of AP65 was localized to the N-terminal region of the protein. In this study we confirmed that cytoadherence to VECs by *T. vaginalis *requires protein synthesis and surface placement of adhesins on trichomonads and showed that AP65 binds to trichomonads, but not trypsin-treated parasites, in an auto-ligand assay. Furthermore, purified recombinant overlapping fragments of AP65 tested for binding activity in a ligand assay showed that a complete N-terminal domain was required for binding to both VECs and parasites. A working model is proposed to explain how this secreted trichomonad protein associates with surface membranes and functions as an adhesin.

## Results

### Binding to VECs requires protein synthesis and surface proteins

**Figure 1:**
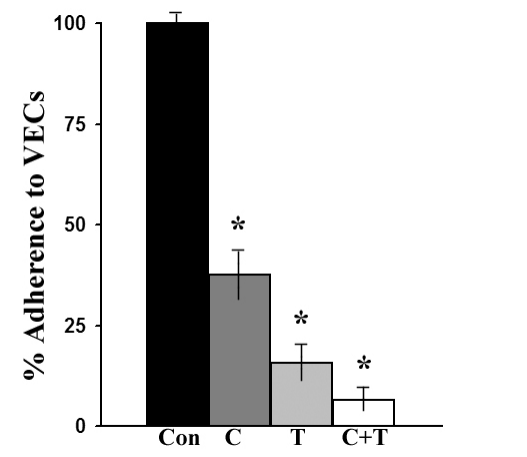
**Representative experiment showing pretreatment of parasites with cycloheximide and/or trypsin abolishes adherence to VECs**. *T. vaginalis *were incubated in the presence or absence of cycloheximide (C), trypsin (T) or the combination of both (C+T) for 30 and 15 min. Treatment and adherence assays were performed as described in Materials and Methods. This graph is a representative experiment of three independent experiments with eight samples per experiment. For each experiment, statistical analysis using the t-test was performed, and error bars indicate the standard deviation for each condition. An asterisk above the bars indicates the p values were all less than 0.05.

We wanted to confirm that cytoadherence to VECs by *T. vaginalis *requires protein synthesis and surface placement of adhesins on trichomonads [14,19,22,29]. We performed an adherence assay with cycloheximide and/or trypsin-treated parasites. As shown in this representative experiment in Figure 1, compared to control, untreated trichomonads (bar Con), inhibition of protein synthesis with cycloheximide for 30 min prior to the adherence assay resulted in a 70% decrease in binding of trichomonads to fixed VEC monolayers (bar C). This result affirms previous data that showed prerequisite synthesis of adhesins for optimal attachment of organisms to host cells [17]. Further, pretreatment of parasites with trypsin alone caused a ~90% decrease in binding (bar T), again affirming involvement of surface proteins in adherence [14,17,22,29]. Treatment with both cycloheximide and trypsin further reduced parasite binding to VECs by 95% (bar C+T). The data for each condition was statistically significant, and as indicated by the asterisks above the bars, all were p < 0.05. The standard deviation for each condition never exceeded 5% of the mean. These treatments did not affect parasite viability and motility. These data show that both de novo synthesis of proteins coupled with surface placement is necessary for optimal attachment to host cells by trichomonads [17,18].

### AP65 associates with fixed T. vaginalis organisms

**Figure 2:**
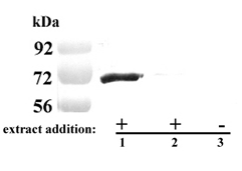
**Representative immunoblot showing native AP65 binds to fixed parasites**. Control, untreated *T. vaginalis *(lane 1) and trypsin-treated parasites (lane 2) were fixed and incubated with detergent lysate of *T. vaginalis *isolate T016 (extract) for 2 h at RT. After extensive washing, bound protein was released from the fixed organisms by boiling in electrophoresis detergent buffer [41] and subjected to SDS-PAGE in an 8% acrylamide gel prior to blotting onto nitrocellulose. Blots were probed with mAb DM116 to AP65. Exogenous AP65 bound to control, untreated parasites (lane 1) but not trypsin-treated organisms (lane 2). As a control, fixed parasites were also subjected to boiling in detergent and handled identically to show no AP65 reactive protein was released under the same conditions (lane 3).

Fluorescence with non-permeabilized trichomonads and immunocytochemical experiments using polyclonal and monoclonal antibody (mAb) detect AP65 on the surface [15,18,30]. AP65 is also one of numerous proteins secreted by the parasite [33]. Therefore, we examined the association of exogenous AP65 in a detergent extract to fixed *T. vaginalis *organisms using an auto-ligand assay similar to a ligand assay employed with VECs (Methods). As seen in Figure 2, immunoblots after SDS-PAGE with mAb DM116 detected AP65 from parasite lysate that bound to fixed trichomonads (lane 1). Organisms first pretreated with trypsin to remove surface proteins followed by the auto-ligand assay had no detectable AP65 bound (lane 2). As a control to show that the AP65 band in lane 1 was due to binding of exogenous AP65 with fixed parasites and not due to the release of any AP65 from the fixed trichomonads, an additional experiment was performed. In this case fixed trichomonads alone were boiled followed by SDS-PAGE and immunoblotting. As seen in lane 3, no AP65 or crossreactive protein was detected, showing that the AP65 band seen in lane 1 is from bound exogenous AP65. The absence of bound AP65 on trypsinized parasites strongly indicates a proteinaceous site on the surface to accommodate the exogenously bound AP65. These data further support the idea that AP65 detected by fluorescence and immunocytochemical assays may in part be due to the association of secreted AP65 with the parasite surface.

### AP65 recombinant fragments react with mAb and polyclonal antibodies to AP65

**Figure 3:**
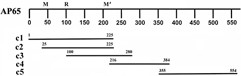
**Diagram representing the AP65-3 recombinant clones**. Clones c1 through c5 represent individual recombinant fragments cloned in pET26b(+) vector (Novagen EMD Chemicals Inc) using primers indicated in Table 1. Each recombinant fragment contains a C-terminal His·Tag^® ^used for purification. The AP65 protein is illustrated above the clones, and numbers indicate the relative positions of amino acids. R is the relative position of rabbit antiserum reactive epitopes, and M and M' refer to epitopes reactive with mAbs 12G4 and F11, respectively, as determined before [18].

**Figure 4:**
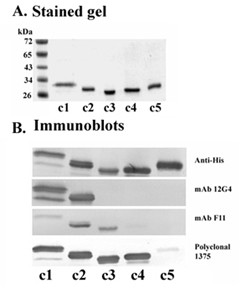
**Expression and antibody reactivity of the purified AP65-3 recombinant subclones shown in Figure 3**. Part A is a Coomassie blue-stained gel of 1 μg purified recombinant proteins after SDS-PAGE using 10% acrylamide gels. The proteins were purified with HisLink™ Protein Purification Resin. Part B shows immunoblots of the clones of panel A after SDS-PAGE of the recombinant fragments probed with anti-Penta-His IgG mAb antibodies (Anti-His), anti-AP65 mAbs (12G4 and F11) [18], and purified rabbit anti-AP65 IgG antibodies (Polyclonal 1375).

We wanted to determine the receptor-binding epitope interacting with both trichomonads and VECs. Overlapping fragments of the *ap65-3 *gene as illustrated in Figure 3 were cloned in the pET-26b(+) vector that contains a C-terminal His·Tag^® ^and expressed in *E. coli *Tuner™ cells. Figure 4 (part A) shows a Coomassie blue-stained gel of the purified AP65 recombinant clones (lanes c1 through c5), and band intensities indicate equivalent amounts of purified proteins were added to each lane. The same amounts were used for immunoblots after SDS-PAGE in part B, which shows reactivities of the five recombinant proteins detected by various antibodies. The recombinant clones had reactivity according to previously-characterized antibody epitope mapping with the mAbs 12G4 (M) and F11 (M'), as illustrated in Figure 3[18]. Polyclonal 1375 is a newly-generated polyclonal rabbit antiserum to AP65. This data indicates that recombinant proteins were expressed and represent fragments based on the AP65 protein. Further, recombinant proteins are readily detected with antibodies essential for probing in subsequent ligand and auto-ligand assays.

### Recombinant subclone c1 binds to VECs and T. vaginalis

**Figure 5:**
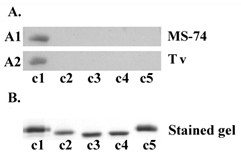
**Recombinant subclone c1 binds to VECS and *T. vaginalis***. Part A shows the immunoblot of chemically-stabilized MS-74 VECs (A1) and *T. vaginalis *(A2) that were incubated with 10 μg of each purified recombinant clone for 1 h at RT. Cells were processed for the ligand assay (Materials and Method). After SDS-PAGE using 10% acrylamide gels, proteins were transferred to PVDF membranes and probed with anti-Penta-His IgG mAb. Only recombinant clone c1 bound to fixed cells in the ligand assay for both cell types. Part B shows a Coomassie-blue stained gel containing 1 μg of each recombinant clones to illustrate that equal amounts of recombinant clones were added to cells in the ligand assays.

In order to identify the receptor-binding domain of AP65, the purified AP65 recombinant protein fragments were used in a ligand assay with MS-74 VECs and *T. vaginalis*. Figure 5 (panel A1) shows results from a representative immunoblot after SDS-PAGE of recombinant protein that bound to MS-74 VECs, and as can be seen, clone c1 containing the complete amino terminus bound to MS-74 VECs (lane c1). Interestingly, clone c2 that is missing the first 25 amino acids as shown in Figure 3 lost complete binding ability (lane c2). These data indicate that the 25 amino acids of the amino-terminus are essential for a functional receptor-binding epitope for VECs. The other recombinant fragments (c3 through c5) also did not bind to VECs in a ligand assay performed identically as with c1. Part B shows the relative equivalent amounts of protein for each clone used in this experiment, as evidenced by staining the gels after SDS-PAGE. This amount visualized in stained gels (part B) represented one-tenth that added to VECs in the ligand assays. In the course of this experiment we performed an auto-ligand assay with fixed *T. vaginalis *organisms followed by SDS-PAGE and immunoblotting. The same amount of each clone as added to VECs was incubated with trichomonads. Remarkably, as with VECs and shown in panel A2, only recombinant protein fragment c1 bound to trichomonads. This indicates that the same recombinant fragment c1 of AP65 bound to both *T. vaginalis *organisms and VECs.

### Recombinant subclone c1 competes with AP65 binding

**Figure 6:**
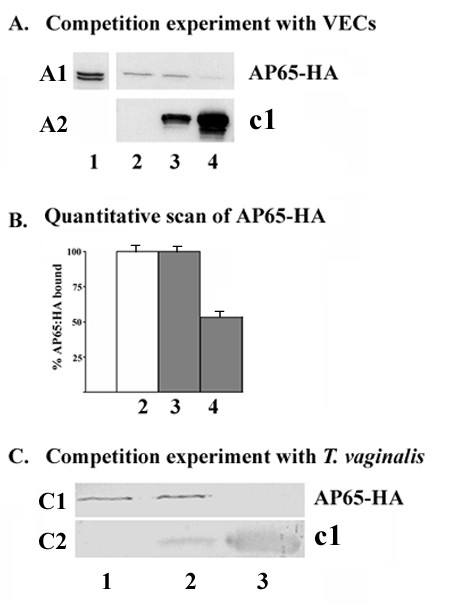
**Clone c1 competes with AP65-HA for binding**. Part A (panel A1) is an immunoblot of a ligand assay of MS-74 VECs pretreated with c1 recombinant protein for 30 min at RT followed by the addition of trichomonad lysate containing AP65-HA and incubated for 1 h. After SDS-PAGE using 8% acrylamide gels and immunoblotting, membranes were probed with anti-HA mAb to detect bound AP65-HA. Purified rabbit anti-AP65 polyclonal IgG (Figure 4) was used to detect c1. Lane 1 is control detergent lysate of T016 isolate expressing AP65-HA showing wild type AP65 (lower band) and the episomally-expressed fusion AP65-HA (upper band). Lane 2 is control VECs without pretreatment with c1. Lanes 3 and 4 are VECs pretreated with 1 μg and 10 μg of c1, respectively. There is visible inhibition of binding of AP65-HA by pretreatment with 10 μg c1. Panel A2 is an immunoblot to detect clone c1 from the same experiment of A1 to show the binding of the recombinant clone c1 to VECs. Part B is a densitometric scan of the AP65-HA immunoblot bands from part A1 to visually present quantitatively the relative percentage of bound AP65-HA. Error bars indicate standard deviations. Competition with 10 μg was significant (p < 0.05). Panel C1 is an immunoblot of a ligand assay of pretreated *T. vaginalis *with c1 recombinant protein and handled identically as in part A. Trichomonads were incubated with 1 μg (lane 2) and 10 μg (lane 3) clone c1 before adding extract with AP65-HA. Lane 1 is untreated, control trichomonads without c1 addition. After SDS-PAGE and immunoblotting, AP65-HA was detected with mAb to HA. Panel C2 detects clone c1 bound to parasites as in A2.

Finally, since recombinant AP65 competes with the natural AP65 for binding to host cells [31], we performed a competition experiment using clone c1 and AP65-HA fusion protein in a ligand assay. In this experiment, fixed MS-74 VECs were pre-incubated with recombinant clone c1 followed by addition of detergent extract derived from transfected parasites episomally expressing AP65-HA [23]. As seen in Figure 6 (panel A1) recombinant clone c1 prevented the binding of AP65-HA in a concentration-dependant manner (lane 4). Densitometric scanning of these same AP65-HA immunoblot bands (panel B) for comparison showed a 50% reduction of bound AP65-HA in VECs pretreated with 10 μg c1 (bar 4). Scans never varied more than 4% of the mean. A decrease by 50% was statistically significant (p < 0.05). Pretreatment with recombinant clone c2 at the same concentrations had no effect (data not shown). Likewise, competition was observed when fixed *T. vaginalis *were used (panel C1, lane 3) and binding of exogenous AP65-HA was fully blocked by pretreatment of fixed parasites with 10 μg of clone c1. Overall, these results indicate strongly that the binding epitope is localized to the N-terminal region of AP65, and the first 25 amino acids of the protein are essential for AP65 binding to both organisms and VECs. Panel A2 and C2 show the clone c1 bound to VECs and organisms, respectively, in these competition experiments.

## Discussion

Binding of *T. vaginalis *to host cells requires synthesis and surface expression of adhesins (Figure 1), in accordance with earlier studies [14,17]. Recent data shows contact with VECs results in the up-regulation of various trichomonad proteins, among which include the trichomonad adhesins [17,20]. The de novo synthesis of proteins might include not only the adhesins but chaperons or vesicle-associated proteins required to transport the adhesins to the surface. In fact, it has been shown that trichomonad disulfide isomerase (PDI), a protein with multiple functions and known to be a chaperon, is also up-regulated upon contact with VECs possibly helping in the compartmentalization of AP65 to various sites [20]. Moreover, that AP65 was found to be secreted by *T. vaginalis *[33] requires that we reassess the manner in which this protein asserts its adhesive function. We hypothesized that AP65 and possibly other adhesins represent surface-associated enzymes bound after secretion and that possess alternative functions as has been shown for other microbial pathogens [34,35]. It is noteworthy, however, that re-association of secreted AP65 for function need not exclude alternative mechanisms of surface placement of AP65. For example, two transmembrane domains have been identified [18], which may play a role for localizing AP65 onto the surface. If in fact there are multiple mechanisms at work for placing AP65 on the parasite surface, then this may represent an adaptation that enhances the overall functions of the adhesin-enzyme.

This data suggests the first 25 amino acids are essential to the binding domain of AP65 that is localized at the amino-terminus of the protein (Figure 5). Deletion of the first 25 amino acids resulted in the complete abolishment of binding to chemically-stabilized cells using a standardized ligand assay [15,18,29] of the recombinant clones (Figure 5). It is possible that a secondary structure formed by these amino acids is responsible for AP65 association. This hypothesis is supported by several observations. Two synthetic peptides (1–16 and 14–30) comprising the first 30 amino acids of AP65 did not inhibit adherence (Materials and Methods; data not shown), and bioinformatic analysis (The PSIPRED Protein Structure Prediction Server, University College London) of AP65 predicted two small helixes are formed by amino acids 8 to 11 and by amino acids 25 to 28. As a result, the recombinant AP65 subclones lacking the 25 amino acids would be missing the first helix, and this likely would yield improper folding of the putative binding motif. In addition, the hydrophobic nature of regions of AP65 [18] may have no role in the binding of AP65 to host cells and parasites [36], and this is illustrated by the fact that all clones possess hydrophobic amino acids. Yet, only one recombinant clone bound to cell surfaces. Further, the recombinant clone c1 contains the 11–12 amino acid hydrogenosomal presequence, and our recent findings suggest based on electrophoretic mobility that the secreted AP65 is a preprotein with the presequence intact [33] and not processed as is the enzymatic form found within hydrogenosomes. This is a significant finding because it permits us understand for the first time the difference between the hydrogenosomal and the surface-associated AP65.

**Figure 7:**
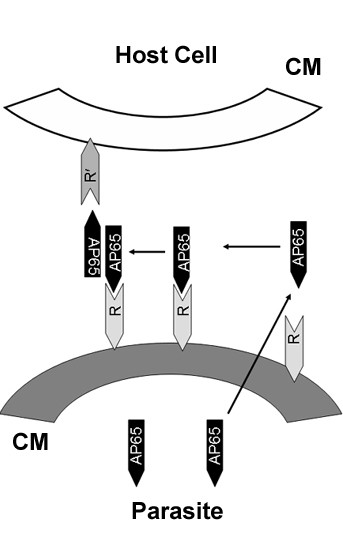
**Model representing the association of secreted AP65 with membranes**. AP65 is secreted to the extracellular environment and re-associates on the parasite surface. This figure shows a protein site on the parasite (labeled R) to which AP65 binds. It is probable that AP65 can re-associate as a monomer or a dimer. In the case of the AP65-dimer one amino-terminus is available to interact with the protein site on the parasite (R) and the second one with an equivalent host cell site (labeled R') forming a bridge that promotes adherence.

A specific interaction between AP65 and a surface structure in both organism and host cells is demonstrated by the inhibition of binding of AP65-HA fusion protein by the N-terminal recombinant c1 fragment (Figure 6) and the association of AP65 with untreated *T. vaginalis*, but not with trypsinized parasites, in the auto-ligand assay (Figure 2). Moreover, the AP65 N-terminal domain binding to both VECs and *T. vaginalis *suggests that such a structure (receptor) has common features in both host cells and parasites. This suggests that the AP65 amino-terminus domain is external on the protein in order to face the surface of both organisms and host cells. Indeed, models of the quaternary structure of malic enzymes show a tetrameric protein with a double dimer structure [37]. The monomer has the N- and C-termini close to each other and protruding outward, thereby making the amino terminal domain exposed for binding. The malic enzyme dimer that is formed by two monomers mirroring each other would provide a basis for binding between parasite and host cell, thus conferring the property of adherence to the organism. Indeed, in the dimer form the N- and C-termini of each malic enzyme monomer do not interact with each other [37]. It is conceivable, therefore, that each AP65 surface dimer would have one amino-terminus exposed that interacts with the parasite and a second one interacting with the host membrane as is proposed by us in the model presented in Figure 7. Furthermore, given the dimeric nature of AP65 with accessible N- termini, it stands to reason that a feature of trichomonads in suspension would be auto-agglutination. Indeed, this is exactly what is seen among fresh clinical isolates, and immunofluorescence with anti-AP65 antibody always detects AP65 parasite-parasite, membrane-membrane regions [15]. Interestingly, laboratory-adapted batch culture trichomonads unable to up-regulate expression of adhesins and that synthesize significant lower amounts of adhesins, including AP65 [29], display little or no self-agglutination.

As mentioned above, housekeeping enzymes of pathogenic organisms are secreted and are anchorless, surface-associated proteins [34,35]. Interestingly, the mechanism of secretion of some of these enzymes is unknown, and enzymes have no secretion peptide signal or LPXTG anchor motif [34,35]. *T. vaginalis *in fact secretes numerous metabolic enzymes, including glyceraldehyde-3-phosphate dehydrogenase, α-enolase, fructose-1-6-biphosphate, and the adhesin AP65 into the extracellular environment [33]. Like bacterial anchorless, surface proteins, *T. vaginalis *secreted metabolic enzymes do not possess any known secretion peptide signal(s) or anchor motif [33]. These characteristics clearly categorize trichomonad AP65 and possibly other secreted enzymes as members of multifunctional proteins similar to those of other microorganisms.

## Conclusion

The secretion and the absence of a covalent anchor motif suggest that AP65 is released extracellularly and binds to the surfaces of both organisms and VECs. Data suggests that the first 25 amino acids of the amino terminus are essential for the receptor-binding epitope. Once bound to a putative receptor on parasites, AP65 could form at least a dimer, since hydrogenosomal decarboxylating malic enzyme-AP65 is a dimer and a tretramer [38] possessing two amino-termini available for binding to respective parasite and host cell. This work permits us to generate a model for the association of trichomonad AP65 with surfaces of both trichomonad and VECs (Figure 7). This model is supported by previous data showing that polyclonal antibodies recognize surface AP65 and prevent cytoadherence [15]. Furthermore, extracellular malic decarboxylase activity identical to AP65 is present [39]. These data collectively support the idea of a protein motif, in both VECs and *T. vaginalis*, binding the N-terminal region of AP65. Further work will confirm the structure and orientation of the AP65 on the surface of *T. vaginalis*.

## Methods

### Parasites and MS74 cell cultures

*T. vaginalis *isolate T016 was grown at 37°C in trypticase-yeast-maltose (TYM) medium supplemented with 10% heat-inactivated horse serum (HS) [40]. T016 isolate expressing AP65-Hemagglutinin (HA) fusion protein was generated by transfection with the pBS-*ap65-3-HA-neo *plasmid, as previously described [16,23]. Stable transfectants were grown at 37°C overnight (o/n) in TYM-10% HS containing Geneticin (100 μg/lm) (GIBCO Invitrogen, Carlsbad, CA). Only mid-logarithmic phase parasites were used throughout.

Primary immortalized MS-74 vaginal epithelial cells (VECs) [18] were grown at 37°C in a 5% CO2 atmosphere in Dulbecco's Modified Eagle Medium (DMEM, GIBCO Invitrogen) supplemented with 10% HI fetal bovine serum (FBS), as before [18]. For the adherence assay, 5 × 10^4 ^MS-74 cells were seeded into each well of 96-well black, clear bottom culture plates (Corning Incorporated Costar^®^, Corning, NY) and grown o/n in DMEM-10% FBS. Cells of a confluent monolayers were washed prior to fixation with 3% glutaraldehyde for 1 h at RT. Monolayers were washed twice and blocked o/n in 0.5 M glycine. Finally, wells were washed twice and maintained in 100 μl of RINGER buffer (0.12 M NaCl, 3.5 mM KCl, 2 mM CaCl_2_, 2.5 mM NaHCO_3_, pH 7.2–7.4) until use.

### Adherence assay

For adherence assays, *T. vaginalis *organisms were grown o/n in TYM-10% HS. Parasites were washed and suspended in TYM for labeling as before [21] prior to any treatment by adding calcein (2 μl ml^-1^) (Molecular Probes™ Invitrogen, Carlsbad, CA). Then, trichomonads were washed in RINGER buffer and suspended to 1 × 10^6 ^parasites ml^-1 ^in TYM containing cycloheximide (20 μg ml^-1^) (Sigma-Aldrich, St. Louis, MO). Cycloheximide treatment of parasites was at 37°C for 30 min. Duplicate cultures of 1 × 10^6 ^parasites ml^-1 ^in TYM were treated with bovine pancreatic type XI trypsin (1 mg ml^-1^) for 15 min at 37°C. Finally, a triplicate preparation of 1 × 10^6 ^organisms ml^-1 ^was treated with cycloheximide (20 μg ml^-1^) for 30 min. However, at the 15 min incubation time point with cycloheximide, trypsin (1 mg ml^-1^) was added and parasites were incubated for the remaining 15 min at 37°C. Treated parasites were washed in TYM and resuspended in TYM to 2.5 × 10^6 ^cells ml^-1^. Then, 100 μl containing 2.5 × 10^5 ^parasites was added to individual wells of 96-well culture plates containing fixed confluent MS-74 VEC monolayers. After incubation for 30 min at 37°C, wells were washed 3 times in RINGER buffer followed by final addition of 200 μl of RINGER buffer. Fluorescence readings were taken at 485/528 nm (excitation/emission) in a Synergy HT plate reader (BioTek Instruments, Inc., Winooski, VT).

For experiments on inhibition of adherence by synthetic peptides, custom peptides were purchased from Sigma-Aldrich-Genosys. The peptides contained the amino-terminus of AP65-3 with the amino acids sequence MLASSVAAPVRNICRA and CRAKLPALKTGMTLLQD. A control peptide was also generated with random amino acids (RLAEVKGGPPHTSDMCNWI). Synthetic peptides were solubilized in 100% dimethylsulfoxide (DMSO) (Sigma-Aldrich), and the peptide concentration was quantified with BCA™ Protein Assay Kit (PIERCE, Rockford, IL). Synthetic peptides were diluted in 100 mM HEPES (Sigma-Aldrich), pH 7.5, to a stock concentration of 100 μg ml^-1^. Then 0.1 ng, 1 ng and 10 ng were added to individual wells of 96-well culture plates containing fixed 100% confluent MS-74 VECs monolayers. Each condition involved quadruplicate samples. Plates were incubated for 1 h at 37°C followed by addition of 100 μl of calcein-labeled parasites (2.5 × 10^5^) to individual wells. After incubation for 20 min at 37°C, plates were washed 3 times in RINGER buffer and handle as above.

### Cloning of trichomonad AP65 recombinant fragments

*T. vaginalis ap6-3 *overlapping gene fragments were amplified by PCR using primers containing 5' and 3' (Table 1) *BglII *restriction site. PCR products were digested and purified using a gel extraction kit (Qiagen, Inc., Valencia, CA). PCR products were ligated into the vector pET-26b(+) (Novagen EMD Chemicals Inc, La Jolla, CA) at a *BamHI *cloning site. This vector contained a Hexa-His·Tag^® ^at the C-terminus for purification of the recombinant protein fragments. Insert direction was verified by PCR using the 5' end primers and the T7 terminator universal primer (data not shown). DH5α *E. coli *was transformed with each individual plasmid by electroporation performed at 2500 V, 25 microfarads and 1000 ohms, using ECM 630 Electro cell manipulator (BTX^®^, Genetronics, Inc., San Diego, CA) in 0.2 cm gap Gene Pulser^® ^Cuvettes (BioRad Laboratories, Hercules, CA). Plasmids were purified using the mini prep plasmid purification kit (Qiagen, Inc.).

**Table 1:** Primers^1 ^used for cloning of *ap65-3 *gene

Clone	Primer sequence for AP65-3	Plasmid
5' c1 Bgl II	GGGGAGATCTGATGCTCGCATCTTCAGTCG	pET-26b(+)
3' c1 Bgl II	GGGGAGATCTGGCCAGCCGTGGTAGAGTG	pET-26b(+)
5' c2 Bgl II	GGGGAGATCTGATGACCCTCCTTCAGG	pET-26b(+)
3' c2 Bgl II	GGGGAGATCTGGCCAGCCGTGGTAGAGTG	pET-26b(+)
5' c3 Bgl II	GGGGAGATCTGGGGAGATTCCTCTTCACACA	pET-26b(+)
3' c3 Bgl II	GGGGAGATCTGGGAAGCAGTTGCAGCGCCAG	pET-26b(+)
5' c4 Bgl II	GGGGAGATCTAATCCTCGCCGACCCA	pET-26b(+)
3' c4 Bgl II	GGGGAGATCTACACCCTCAAGGACGGAG	pET-26b(+)
5' c5 Bgl II	GGGGAGATCTGATGGTCCATGCTGACCGTA	pET-26b(+)
3' c5 Bgl II	GGGGAGATCTAGGTCCTTGAGCTCTGTG	pET-26b(+)
5' FL NdeI	GTCCAGCATATGATGCTCGCATCTTCAGTCG	pBS-*ap65-HA*
3' FL Asp718	GTCCACGGTACCATAGTAGAGTTGCTCGTATTC	pBS-*ap65-HA*

^1 ^These primers were used for the cloning of overlapping fragments and full-length of the *ap65-3 *gene in the pET-26b(+) vector (Novagen EMD Chemicals Inc) in *E. coli *and pBS-HA vector in *T. vaginalis *[16, 23].

### Expression and purification of recombinant AP65 fragments

*E. coli *Tuner™ (DE3) competent cells (Novagen EMD Chemicals Inc) were transformed with individual AP65 construct plasmids and selected with kanamycin. Single colonies were used for culture and batch protein purification. One hundred ml of LB containing 50 μg ml^-1 ^kanamycin were inoculated with 10 ml of o/n cultures of *E. coli *encoding each recombinant AP65 fragments. Cultures were grown for 3 h at 37°C with shaking. Induction of expression of recombinant protein was done by adding 0.4 mM isopropyl-β-D-thiogalactopyranoside (IPTG) (Fisher Scientific, Pittsburgh, PA), and the cultures were incubated for an additional 2.5 h at 37°C with shaking. Pellets were collected and bacteria lysed with 10 ml lysis buffer (50 mM sodium phosphate buffer, 50 mM Tris HCl, pH 8.0, 0.3 M NaCl, 1% Triton ×-100, 1% deoxycholic acid, and 8 M urea), sonicated for 2–5 min, and incubated for 30 min at 37°C. Debris in the lysate was clarified by centrifugation at 13,000 rpm for 30 min at 4°C in a Sorvall^® ^SS-34 rotor (Thermo Fisher Scientific, Inc. Waltham, MA). Purification of recombinant proteins was done using HisLink™ Protein Purification Resin (Promega, Madison, WI), according to the manufacture's protocol. Briefly, lysate was added to 1 ml of resin and allowed to bind for 1 h at 4°C. Resin was allowed to precipitate and washed twice with 100 mM HEPES, pH 7.5, 25 mM imidazole, 8 M urea, and once with 100 mM HEPES, pH 7.5, 50 mM imidazole, and 8 M Urea. Finally, recombinant protein was obtained by placing the resin in a glass column. The bound recombinant proteins were eluted with 3 ml of 100 mM HEPES, pH 7.5, 750 mM imidazole, and 8 M urea. Purified recombinant proteins were dialyzed to remove the urea in Slide-A-Lyzer^® ^dialysis cassettes (PIERCE,) with a 10,000 MW cutoff in a stepwise procedure. Cassettes were placed in a dialysis buffer (50 mM sodium bicarbonate, 1 mM DTT, 1 mM EDTA) containing decreasing concentrations of urea as follows: i) 500 ml dialysis buffer, pH 9.0, with 4 M urea, for 1 h at RT, ii) 500 ml dialysis buffer, pH 9.0, 2 M urea, for 2 h at 4°C, with change of buffer after an hour, iii) 200 ml dialysis buffer, pH 8.3, for 1 h at 4°C, without urea, and, iv) 500 ml dialysis buffer, pH 9.0, o/n at 4°C without urea. Protein concentration was quantified with the BCA™ Protein Assay Kit (PIERCE) and by SDS-PAGE.

### Ligand assay and immunoblotting

This ligand assay has been extensively described [15,18,29]. MS-74 VECs were washed in RINGER buffer and fixed in 3% glutaraldehyde for 30 min at RT. Cells were washed twice and blocked o/n in 0.5 M glycine. For the ligand assay using recombinant AP65 subclones c1 through c5 (Figure 5, part A1), 10 μg of each purified recombinant protein was added to 2 × 10^5 ^fixed MS-74 VECs and allowed to bind for 1 h at RT.

For the auto-ligand assay, the experiment was performed using fixed trichomonads (Figure 5, part A2). In this case, 2 × 10^6 ^parasites were incubated with each clone identical to the ligand assay described with VECs. For trypsinized parasites (Figure 2, lane 2), 1 × 10^7 ^parasites ml^-1 ^were first treated with trypsin (10 mg ml^-1^) for 15 min at 37°C before fixation. In addition, the auto-ligand assay was performed using lysate generated from 1 × 10^7 ^T016 parasites, as previously described [15]. This detergent extract was incubated with 2 × 10^6 ^fixed *T. vaginalis *for 2 h at RT.

For competition studies with recombinant clones (Figure 6), fixed MS-74 VECs (2 × 10^5^) and *T. vaginalis *(2 × 10^6^) were incubated with 1 μg and 10 μg of recombinant AP65 clones c1 and c2 for 30 min at RT. Lysate from 1 × 10^7 ^T016 expressing the AP65-HA fusion protein was prepared as before [15], and protein concentration was determined with the BCA™ Protein Assay Kit (PIERCE). Then 10 μg of protein lysate were added to pretreated MS-74 VECs or *T. vaginalis *and incubated for an additional h at RT. After incubation with lysate or recombinant protein, cells were washed 3 times, and bound protein was solubilized by boiling for 3 min in 30 μl of 2× SDS electrophoresis sample buffer [41]. Protein was separated on 8% or 10% acrylamide SDS-PAGE and transferred to nitrocellulose or PVDF membranes (BioRad Laboratories) for immunoblotting. Membranes were probed with mAb to AP65 [18] and polyclonal anti-AP65 serum IgG antibodies, IgG mAb to Penta-His (Qiagen, Inc.), and IgG mAb to HA (Sigma-Aldrich). After o/n incubation with primary antibodies, the blots were extensively washed and incubated for 1 to 2 h in either secondary alkaline phosphatase- (AP) or horseradish peroxidase-conjugated goat anti-mouse IgG (Sigma-Aldrich) or AP-conjugated goat anti-rabbit IgG (BioRad Laboratories). Both primary and secondary antibodies were used at 1:1000 dilutions.

### Reproducibility of experiments

Unless otherwise stated in the text, all experiments were performed numerous times and no less than on three different occasions. Statistical analysis using the t-test was performed as needed, and as indicated with asterisks, the p values were less than 0.05. Error bars represent standard deviations.

## Abbreviations

AP65, adhesin protein of molecular weight 65-kDa; AP65-HA, fusion protein of AP65 and hemagglutinin (HA), HEPES, N-2-hydroxyethyl piperazine-N' -2-ethanesulfonic acid; mAb, monoclonal antibody; SDS-PAGE, sodium dodecylsulfate polyacrylamide gel electrophoresis; RT, room temperature; Tv, *Trichomonas vaginalis *VEC, vaginal epithelial cell

## Authors' contributions

AFG carried out the design of the study and performed plasmid constructions, transformations, recombinant protein expression and purification, immunoblots, ligand assays, adherence assay and drafted the manuscript. JFA participated in the design of the experiments, offered suggestions during the experiments, and helped to write the manuscript. All the authors have read and approved the manuscript.
